# 3-(4-{3,3,4,4,5,5-Hexafluoro-2-[5-(3-methoxy­phen­yl)-2-methyl-3-thien­yl]cyclo­pent-1-en­yl}-5-methyl-2-thien­yl)benzonitrile

**DOI:** 10.1107/S1600536809034771

**Published:** 2009-09-05

**Authors:** An-yin Chen, Zhang-Gao Le, Gang Liu, Shou-Zhi Pu, Cong-Bin Fan

**Affiliations:** a College of Biology, Chemistry and Material Science, East China Institute of Technology, Fuzhou 344000, People’s Republic of China; b Jiangxi Key Laboratory of Organic Chemistry, Jiangxi Science & Technology Normal University, Nanchang 330013, People’s Republic of China

## Abstract

The title compound, C_29_H_19_F_6_NOS_2_, is a new unsymmetrical photochromic diarylethene derivative with different *meta*-phenyl substituents. The distance between the two reactive (*i.e.* can be irradiated to form a new chemical bond) C atoms is 3.501 (4) Å; the dihedral angles between the mean plane of the main central cyclo­pentene ring and the thio­phene rings are 47.7 (5) and 45.1 (2)°, and those between the thio­phene rings and the adjacent benzene rings are 29.4 (2) and 28.4 (3)°. The three C atoms and the F atoms of hexa­fuorocyclo­pentene ring are disordered over two positions, with site-occupancy factors of 0.751 (4) and 0.249 (4).

## Related literature

For related compounds, see: Irie (2000 ▶); Irie *et al.* (2001 ▶); Pu *et al.* (2007 ▶, 2008 ▶). For the synthesis of the precursors, see: Pu *et al.* (2006 ▶); Yang *et al.* (2007 ▶). For ring-closure reactions, see: Ramamurthy & Venkatesan (1987 ▶).
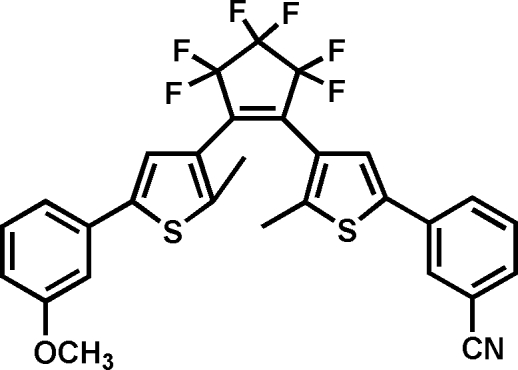


## Experimental

### 

#### Crystal data

C_29_H_19_F_6_NOS_2_
*M* *_r_* = 575.57
Triclinic, 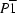

*a* = 8.6134 (11) Å
*b* = 11.5938 (15) Å
*c* = 14.5281 (19) Å
α = 68.346 (2)°
β = 88.783 (2)°
γ = 82.158 (2)°
*V* = 1335.1 (3) Å^3^
*Z* = 2
Mo *K*α radiation
μ = 0.27 mm^−1^
*T* = 296 K
0.15 × 0.13 × 0.10 mm

#### Data collection

Bruker SMART CCD area-detector diffractometer
Absorption correction: multi-scan (**SADABS**; Sheldrick, 1996 ▶) *T*_min_ = 0.961, *T*_max_ = 0.974
10275 measured reflections
4948 independent reflections
3155 reflections with *I* > 2σ(*I*)
*R*_int_ = 0.029

#### Refinement

*R*[*F* ^2^ > 2σ(*F* ^2^)] = 0.049
*wR*(*F* ^2^) = 0.127
*S* = 1.03
4948 reflections
383 parameters
16 restraints
H-atom parameters constrained
Δρ_max_ = 0.30 e Å^−3^
Δρ_min_ = −0.23 e Å^−3^

### 

Data collection: *SMART* (Bruker, 1997 ▶); cell refinement: *SAINT* (Bruker, 1997 ▶); data reduction: *SAINT*; program(s) used to solve structure: *SHELXS97* (Sheldrick, 2008 ▶); program(s) used to refine structure: *SHELXL97* (Sheldrick, 2008 ▶); molecular graphics: *SHELXTL* (Sheldrick, 2008 ▶); software used to prepare material for publication: *SHELXTL*.

## Supplementary Material

Crystal structure: contains datablocks I, global. DOI: 10.1107/S1600536809034771/is2442sup1.cif

Structure factors: contains datablocks I. DOI: 10.1107/S1600536809034771/is2442Isup2.hkl

Additional supplementary materials:  crystallographic information; 3D view; checkCIF report

## Figures and Tables

**Fig. 1.**
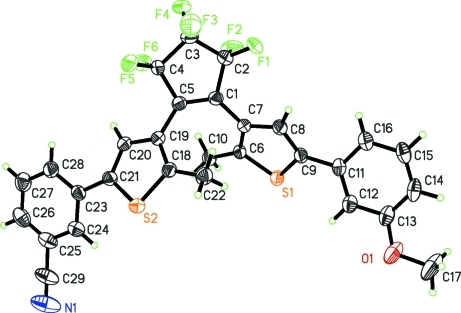
Molecular structure of the title compound; thermal displacement ellipsoids are drawn at the 30% probability level. H atoms are represented as small spheres of arbitrary radius. Only the major component of the disordered groups are shown.

**Fig. 2.**
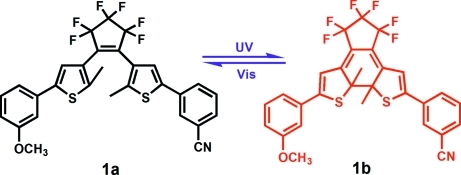
Photochromic interconvertion of the title compound.

## supplementary crystallographic information

### Comment

Photochromic diarylethene is one of the most promising candidates for photoelectronic applications, such as optical storage (Irie, 2000; Pu *et al.*, 2008), photoswitches (Irie *et al.*, 2001; Pu *et al.*, 2007) and waveguides. In the hexafluorocyclophentene ring of the title compound, C1=C5 [1.342 (4) Å] is clearly a double bond, being significantly shorter than the other single bonds, such as C1-C2 [1.502 (6) Å], C2-C3 [1.530 (6) Å], C3-C4 [1.583 (1) Å], C1-C7 [1.467 (4) Å], C5-C19 [1.471 (4) Å]. The title compound shows a photoactive antiparallel conformation (Fig. 1). The two independent planar thiophene ring systems have essentially identical geometries, and the dihedral angles between the main central cyclopent-1-ene ring and those of the two thiophene rings, S1/C6-C9 and S2/C18-C21, are 47.7 (5) and 45.1 (2)°, respectively. The dihedral angle between the thiophene and adjacent benzene ring is 29.4 (2)° for the C11-C16 benzene ring and 28.4 (3)° for the C23-C28 benzene ring. The two thiophene groups are linked by the C1=C5 double bond, with both of them attached to the ethylene group *via*  the 2-position. The distance between the two reactive C atoms (C6···C18) is 3.501 (4) Å, which is short enough, theoretically, for the ring-closure reaction to take place in the crystalline phase (Ramamurthy & Venkatesan, 1987). Crystals of the title compound (1a) show photochromism in accordance with the expected ring closure, to form (1b) (Fig. 2); upon irradiation with 313 nm light, the colorless crystals turn blue rapidly. The blue compound when dissolved in hexane displays an absorption maximum at 583 nm. Upon irradiation with visible light with a wavelength greater than 450 nm, the blue crystals again turn initial colorless; a hexane solution has an absorption maximum at 294 nm.

### Experimental

The title compound was originally derived from 3-bromo-5-(3-methoxyphenyl)-2-methylthiophene (1) (Yang *et al.*, 2007) (5.0 g, 17.6 mmol) with an n-BuLi/hexane solution (7.1 mL, 2.5 M, 17.6 mmol) at 195 K under a nitrogen atmosphere, after 30 min, perfluorocyclopentene (2.2 mL, 17.6 mmol) was added quickly to the reaction solution. The mixture was stirred for 3 h at this temperature. The reaction mixture was filtered and evaporated in vacuo. The residue was purified by column chromatography on silica gel (hexane) to give (2-methyl-5- (3-methoxylphenyl)thiophene)perfluorocyclopent-1-ene, (2) (4.53 g, 11.3 mmol). Finally, to a stirred THF solution (50 ml) of 3-bromo-5-(3-cyanophenyl)-2-methylthiophene, (3) (Pu *et al.*, 2006) (2.6 g, 9.6 mmol), an n-BuLi/hexane solution (3.8 ml, 2.5 M, 9.6 mmol) was slowly added in at 195 K under a nitrogen atmosphere. After 30 min, compound (2) (3.8 g, 9.6 mmol) was added and the mixture was stirred for 2 h at this temperature. The reaction mixture was extracted with diethyl ether and evaporated in vacuo, then purified by column chromatography (petroleum ether) to give the title compound, (Ia) (2.41 g, 4.2 mmol), in 43.7% yield. ^1^H NMR (400 MHz, CDCl_3_, TMS): δ 1.88 (s, 3H, -CH_3_),1.90 (s, 3H, -CH_3_), 3.76 (s, 3H, -OCH_3_), 6.76-6.78 (dd, 1H, Hz, thiophene-H), 6.97 (s, 1H, thiophene-H), 7.04, 7.06 (d, 1H, J=8.0 Hz, phenyl -H), 7.17-7.24 (m, 2H, phenyl -H), 7.27 (s, 1H, phenyl-H), 7.41 (t, 1H, J=8.0 Hz, phenyl -H), 7.48, 7.50 (d, 1H, J=8.0 Hz, phenyl -H), 7.66, 7.68 (d, 1H, J=8.0 Hz, phenyl -H), 7.72 (s, 1H, phenyl-H); Elemental analysis: calc. for C_29_H_19_F_6_NOS_2_: C 60.20, H 3.83%. Found: C 58.84, H 3.77%; m.p.: 369.4–370.8 K.

### Refinement

All H atoms attached to C were geometrically positioned and treated as riding with C—H = 0.96 Å (methyl) or 0.93 Å (aromatic), and with *U*_iso_(H) = 1.2*U*_eq_(aromatic C) or 1.5*U*_eq_(methyl C). The cyclopent-1-ene ring in C2, C3, C4-envelope conformation is disorder. The occupancies of the disorder components were refined to 0.751 (4):0.249 (4) for C2:C2', C3:C3', C4:C4' and  F1:F1', F2:F2', F3:F3', F4:F4', F5:F5', F6:F6'. The disordered atoms were refined with bond restraints of C—F = 1.34 (1) and C—C = 1.50 (1) Å, and with constraints of same displacement parameters for major and minor atoms.

### Crystal data

**Table:**

C_29_H_19_F_6_NOS_2_	*Z* = 2
*M**_r_* = 575.57	*F*(000) = 588
Triclinic, *P*1	*D*_x_ = 1.432 Mg m^−^^3^
*a* = 8.6134 (11) Å	Mo *K*α radiation, λ = 0.71073 Å
*b* = 11.5938 (15) Å	Cell parameters from 1963 reflections
*c* = 14.5281 (19) Å	θ = 2.4–21.8°
α = 68.346 (2)°	µ = 0.27 mm^−^^1^
β = 88.783 (2)°	*T* = 296 K
γ = 82.158 (2)°	Block, colourless
*V* = 1335.1 (3) Å^3^	0.15 × 0.13 × 0.10 mm

### Data collection

**Table:**

Bruker SMART CCD area-detector diffractometer	4948 independent reflections
Radiation source: fine-focus sealed tube	3155 reflections with *I* > 2σ(*I*)
graphite	*R*_int_ = 0.029
φ and ω scans	θ_max_ = 25.5°, θ_min_ = 2.4°
Absorption correction: multi-scan (*SADABS*; Sheldrick, 1996)	*h* = −10→10
*T*_min_ = 0.961, *T*_max_ = 0.974	*k* = −14→14
10275 measured reflections	*l* = −17→17

### Refinement

**Table:**

Refinement on *F*^2^	Primary atom site location: structure-invariant direct methods
Least-squares matrix: full	Secondary atom site location: difference Fourier map
*R*[*F*^2^ > 2σ(*F*^2^)] = 0.049	Hydrogen site location: inferred from neighbouring sites
*wR*(*F*^2^) = 0.127	H-atom parameters constrained
*S* = 1.03	*w* = 1/[σ^2^(*F*_o_^2^) + (0.0556*P*)^2^ + 0.1414*P*] where *P* = (*F*_o_^2^ + 2*F*_c_^2^)/3
4948 reflections	(Δ/σ)_max_ < 0.001
383 parameters	Δρ_max_ = 0.30 e Å^−^^3^
16 restraints	Δρ_min_ = −0.22 e Å^−^^3^

### Special details

**Table:**

Geometry. All esds (except the esd in the dihedral angle between two l.s. planes) are estimated using the full covariance matrix. The cell esds are taken into account individually in the estimation of esds in distances, angles and torsion angles; correlations between esds in cell parameters are only used when they are defined by crystal symmetry. An approximate (isotropic) treatment of cell esds is used for estimating esds involving l.s. planes.
Refinement. Refinement of F^2^ against ALL reflections. The weighted R-factor wR and goodness of fit S are based on F^2^, conventional R-factors R are based on F, with F set to zero for negative F^2^. The threshold expression of F^2^ > 2sigma(F^2^) is used only for calculating R-factors(gt) etc. and is not relevant to the choice of reflections for refinement. R-factors based on F^2^ are statistically about twice as large as those based on F, and R- factors based on ALL data will be even larger.

### Fractional atomic coordinates and isotropic or equivalent isotropic displacement parameters (Å^2^)

**Table:**

	*x*	*y*	*z*	*U*_iso_*/*U*_eq_	Occ. (<1)
C2	0.8607 (7)	0.8219 (8)	0.1970 (5)	0.0616 (8)	0.751 (4)
C3	0.9550 (6)	0.8673 (5)	0.2620 (5)	0.0653 (13)	0.751 (4)
C4	0.8293 (11)	0.9522 (4)	0.2991 (4)	0.0510 (14)	0.751 (4)
F1	0.9173 (6)	0.7019 (6)	0.2107 (4)	0.0911 (14)	0.751 (4)
F2	0.8822 (6)	0.8920 (4)	0.1004 (4)	0.0963 (15)	0.751 (4)
F3	1.0171 (4)	0.7695 (3)	0.3431 (2)	0.1018 (11)	0.751 (4)
F4	1.0726 (3)	0.9286 (4)	0.2184 (3)	0.0965 (11)	0.751 (4)
F5	0.8628 (3)	0.9419 (3)	0.3914 (2)	0.0814 (9)	0.751 (4)
F6	0.8338 (4)	1.0749 (3)	0.2401 (2)	0.0802 (11)	0.751 (4)
C2'	0.8610 (15)	0.822 (2)	0.1990 (15)	0.0616 (8)	0.249 (4)
C3'	0.9453 (17)	0.9039 (17)	0.2351 (15)	0.0653 (13)	0.249 (4)
C4'	0.848 (4)	0.9271 (15)	0.2995 (13)	0.0510 (14)	0.249 (4)
F1'	0.904 (2)	0.700 (2)	0.2492 (14)	0.0911 (14)	0.249 (4)
F2'	0.877 (2)	0.8315 (16)	0.1047 (14)	0.0963 (15)	0.249 (4)
F3'	1.0853 (14)	0.8369 (11)	0.2718 (9)	0.1018 (11)	0.249 (4)
F4'	0.9731 (9)	0.9989 (10)	0.1527 (8)	0.0965 (11)	0.249 (4)
F5'	0.8899 (11)	0.8652 (10)	0.3949 (8)	0.0814 (9)	0.249 (4)
F6'	0.8456 (16)	1.0469 (13)	0.2921 (9)	0.0802 (11)	0.249 (4)
C1	0.6932 (3)	0.8443 (2)	0.2242 (2)	0.0474 (7)	
C5	0.6795 (3)	0.9091 (2)	0.28436 (19)	0.0460 (7)	
C6	0.4380 (3)	0.8805 (2)	0.13185 (19)	0.0451 (6)	
C7	0.5704 (3)	0.8033 (2)	0.1798 (2)	0.0461 (7)	
C8	0.5748 (3)	0.6789 (3)	0.1826 (2)	0.0516 (7)	
H8	0.6580	0.6159	0.2111	0.062*	
C9	0.4467 (3)	0.6608 (2)	0.1399 (2)	0.0482 (7)	
C10	0.3912 (3)	1.0166 (2)	0.1107 (2)	0.0545 (7)	
H10A	0.3333	1.0270	0.1650	0.082*	
H10B	0.3269	1.0533	0.0511	0.082*	
H10C	0.4834	1.0571	0.1024	0.082*	
C11	0.4066 (4)	0.5459 (3)	0.1315 (2)	0.0520 (7)	
C12	0.2517 (4)	0.5282 (3)	0.1271 (2)	0.0593 (8)	
H12	0.1722	0.5894	0.1304	0.071*	
C13	0.2130 (4)	0.4207 (3)	0.1178 (2)	0.0673 (9)	
C14	0.3297 (5)	0.3315 (3)	0.1111 (2)	0.0739 (10)	
H14	0.3046	0.2602	0.1029	0.089*	
C15	0.4834 (5)	0.3476 (3)	0.1163 (2)	0.0688 (9)	
H15	0.5620	0.2861	0.1126	0.083*	
C16	0.5244 (4)	0.4538 (3)	0.1270 (2)	0.0620 (8)	
H16	0.6293	0.4631	0.1310	0.074*	
C17	0.0082 (6)	0.3090 (4)	0.1022 (4)	0.1315 (19)	
H17A	0.0523	0.3022	0.0429	0.197*	
H17B	−0.1042	0.3193	0.0965	0.197*	
H17C	0.0442	0.2343	0.1580	0.197*	
C18	0.4268 (3)	0.8736 (3)	0.37482 (19)	0.0480 (7)	
C19	0.5397 (3)	0.9477 (2)	0.33159 (19)	0.0452 (7)	
C20	0.5058 (3)	1.0699 (3)	0.33379 (19)	0.0480 (7)	
H20	0.5720	1.1306	0.3087	0.058*	
C21	0.3672 (3)	1.0900 (3)	0.3761 (2)	0.0503 (7)	
C22	0.4206 (4)	0.7390 (3)	0.3910 (2)	0.0602 (8)	
H22A	0.3639	0.7343	0.3366	0.090*	
H22B	0.3687	0.7003	0.4515	0.090*	
H22C	0.5253	0.6962	0.3951	0.090*	
C23	0.2932 (3)	1.2076 (3)	0.38317 (19)	0.0511 (7)	
C24	0.1306 (4)	1.2368 (3)	0.3827 (2)	0.0610 (8)	
H24	0.0666	1.1797	0.3799	0.073*	
C25	0.0641 (4)	1.3508 (3)	0.3865 (2)	0.0698 (9)	
C26	0.1566 (5)	1.4367 (3)	0.3904 (2)	0.0796 (10)	
H26	0.1110	1.5126	0.3935	0.095*	
C27	0.3175 (5)	1.4096 (3)	0.3896 (3)	0.0760 (10)	
H27	0.3805	1.4679	0.3912	0.091*	
C28	0.3851 (4)	1.2964 (3)	0.3863 (2)	0.0619 (8)	
H28	0.4937	1.2789	0.3863	0.074*	
C29	−0.1043 (5)	1.3799 (4)	0.3840 (3)	0.0919 (13)	
N1	−0.2356 (4)	1.4047 (4)	0.3815 (3)	0.1329 (16)	
O1	0.0558 (3)	0.4145 (2)	0.1161 (2)	0.0975 (9)	
S1	0.32016 (9)	0.79945 (7)	0.09174 (5)	0.0523 (2)	
S2	0.28003 (9)	0.95549 (7)	0.41707 (6)	0.0570 (2)	

### Atomic displacement parameters (Å^2^)

**Table:**

	*U*^11^	*U*^22^	*U*^33^	*U*^12^	*U*^13^	*U*^23^
C2	0.0492 (19)	0.066 (2)	0.075 (2)	0.0006 (16)	0.0011 (16)	−0.0354 (18)
C3	0.036 (2)	0.067 (4)	0.096 (5)	0.000 (2)	−0.005 (2)	−0.035 (3)
C4	0.048 (3)	0.050 (3)	0.063 (2)	−0.008 (3)	0.0055 (16)	−0.0309 (19)
F1	0.0546 (17)	0.0930 (16)	0.155 (5)	0.0099 (12)	0.006 (3)	−0.087 (3)
F2	0.0647 (14)	0.153 (5)	0.0747 (16)	−0.029 (3)	0.0199 (11)	−0.042 (3)
F3	0.092 (2)	0.095 (2)	0.114 (3)	0.0270 (18)	−0.0439 (19)	−0.045 (2)
F4	0.0457 (17)	0.140 (3)	0.145 (3)	−0.0392 (19)	0.0381 (17)	−0.093 (2)
F5	0.0607 (16)	0.130 (3)	0.0804 (16)	−0.0208 (19)	−0.0012 (12)	−0.067 (2)
F6	0.0650 (15)	0.057 (2)	0.115 (3)	−0.0247 (14)	0.024 (2)	−0.023 (2)
C2'	0.0492 (19)	0.066 (2)	0.075 (2)	0.0006 (16)	0.0011 (16)	−0.0354 (18)
C3'	0.036 (2)	0.067 (4)	0.096 (5)	0.000 (2)	−0.005 (2)	−0.035 (3)
C4'	0.048 (3)	0.050 (3)	0.063 (2)	−0.008 (3)	0.0055 (16)	−0.0309 (19)
F1'	0.0546 (17)	0.0930 (16)	0.155 (5)	0.0099 (12)	0.006 (3)	−0.087 (3)
F2'	0.0647 (14)	0.153 (5)	0.0747 (16)	−0.029 (3)	0.0199 (11)	−0.042 (3)
F3'	0.092 (2)	0.095 (2)	0.114 (3)	0.0270 (18)	−0.0439 (19)	−0.045 (2)
F4'	0.0457 (17)	0.140 (3)	0.145 (3)	−0.0392 (19)	0.0381 (17)	−0.093 (2)
F5'	0.0607 (16)	0.130 (3)	0.0804 (16)	−0.0208 (19)	−0.0012 (12)	−0.067 (2)
F6'	0.0650 (15)	0.057 (2)	0.115 (3)	−0.0247 (14)	0.024 (2)	−0.023 (2)
C1	0.0408 (16)	0.0449 (16)	0.0557 (17)	−0.0031 (13)	0.0014 (13)	−0.0187 (14)
C5	0.0428 (16)	0.0451 (15)	0.0501 (16)	−0.0076 (12)	0.0010 (13)	−0.0170 (13)
C6	0.0476 (16)	0.0459 (16)	0.0465 (16)	−0.0076 (13)	0.0049 (13)	−0.0224 (13)
C7	0.0415 (16)	0.0491 (16)	0.0538 (17)	−0.0065 (13)	0.0037 (13)	−0.0261 (14)
C8	0.0479 (17)	0.0475 (17)	0.0603 (18)	0.0008 (13)	−0.0026 (14)	−0.0231 (14)
C9	0.0516 (17)	0.0431 (15)	0.0531 (17)	−0.0030 (13)	0.0019 (13)	−0.0226 (13)
C10	0.0567 (18)	0.0443 (16)	0.0621 (19)	−0.0028 (14)	−0.0032 (14)	−0.0205 (14)
C11	0.066 (2)	0.0456 (16)	0.0459 (16)	−0.0046 (15)	−0.0030 (14)	−0.0198 (13)
C12	0.068 (2)	0.0485 (17)	0.0628 (19)	−0.0063 (15)	−0.0068 (16)	−0.0218 (15)
C13	0.087 (3)	0.0470 (18)	0.068 (2)	−0.0173 (18)	−0.0074 (18)	−0.0186 (16)
C14	0.115 (3)	0.0417 (18)	0.069 (2)	−0.018 (2)	−0.003 (2)	−0.0227 (16)
C15	0.094 (3)	0.0420 (18)	0.068 (2)	0.0048 (18)	0.0031 (19)	−0.0227 (16)
C16	0.075 (2)	0.0513 (18)	0.0618 (19)	−0.0045 (16)	0.0054 (16)	−0.0252 (15)
C17	0.135 (4)	0.084 (3)	0.179 (5)	−0.048 (3)	−0.038 (3)	−0.038 (3)
C18	0.0487 (17)	0.0518 (16)	0.0472 (16)	−0.0107 (13)	0.0008 (13)	−0.0212 (14)
C19	0.0427 (16)	0.0483 (16)	0.0472 (16)	−0.0083 (13)	0.0006 (13)	−0.0200 (13)
C20	0.0450 (17)	0.0511 (17)	0.0520 (16)	−0.0127 (13)	0.0014 (13)	−0.0218 (14)
C21	0.0439 (17)	0.0579 (18)	0.0505 (16)	−0.0038 (14)	−0.0013 (13)	−0.0228 (14)
C22	0.068 (2)	0.0564 (18)	0.0583 (18)	−0.0188 (16)	0.0079 (15)	−0.0204 (15)
C23	0.0500 (17)	0.0582 (18)	0.0454 (16)	0.0004 (14)	−0.0003 (13)	−0.0221 (14)
C24	0.056 (2)	0.067 (2)	0.0564 (18)	−0.0040 (16)	0.0048 (15)	−0.0202 (16)
C25	0.061 (2)	0.070 (2)	0.062 (2)	0.0079 (18)	0.0113 (16)	−0.0133 (18)
C26	0.090 (3)	0.062 (2)	0.078 (2)	0.018 (2)	0.005 (2)	−0.0260 (19)
C27	0.087 (3)	0.059 (2)	0.086 (2)	−0.0044 (19)	−0.002 (2)	−0.0330 (19)
C28	0.059 (2)	0.063 (2)	0.067 (2)	−0.0024 (16)	−0.0043 (16)	−0.0303 (17)
C29	0.067 (3)	0.087 (3)	0.091 (3)	0.017 (2)	0.022 (2)	−0.008 (2)
N1	0.078 (3)	0.123 (3)	0.143 (3)	0.021 (2)	0.034 (2)	0.001 (2)
O1	0.096 (2)	0.0707 (16)	0.129 (2)	−0.0306 (15)	−0.0231 (17)	−0.0324 (16)
S1	0.0505 (4)	0.0490 (4)	0.0619 (5)	−0.0042 (3)	−0.0044 (3)	−0.0264 (4)
S2	0.0502 (5)	0.0664 (5)	0.0616 (5)	−0.0165 (4)	0.0121 (4)	−0.0295 (4)

### Geometric parameters (Å, °)

**Table:**

C2—F1	1.352 (5)	C12—C13	1.386 (4)
C2—F2	1.361 (6)	C12—H12	0.9300
C2—C1	1.502 (6)	C13—O1	1.367 (4)
C2—C3	1.530 (6)	C13—C14	1.370 (5)
C3—F4	1.331 (5)	C14—C15	1.370 (5)
C3—F3	1.354 (5)	C14—H14	0.9300
C3—C4	1.583 (11)	C15—C16	1.388 (4)
C4—F5	1.337 (5)	C15—H15	0.9300
C4—F6	1.369 (5)	C16—H16	0.9300
C4—C5	1.492 (8)	C17—O1	1.424 (4)
C2'—F2'	1.340 (10)	C17—H17A	0.9600
C2'—F1'	1.339 (10)	C17—H17B	0.9600
C2'—C1	1.494 (10)	C17—H17C	0.9600
C2'—C3'	1.505 (10)	C18—C19	1.372 (4)
C3'—F4'	1.338 (10)	C18—C22	1.499 (4)
C3'—F3'	1.345 (10)	C18—S2	1.716 (3)
C3'—C4'	1.32 (4)	C19—C20	1.420 (4)
C4'—F5'	1.334 (10)	C20—C21	1.362 (4)
C4'—F6'	1.350 (10)	C20—H20	0.9300
C4'—C5	1.53 (3)	C21—C23	1.462 (4)
C1—C5	1.342 (4)	C21—S2	1.722 (3)
C1—C7	1.467 (4)	C22—H22A	0.9600
C5—C19	1.471 (4)	C22—H22B	0.9600
C6—C7	1.371 (4)	C22—H22C	0.9600
C6—C10	1.490 (3)	C23—C28	1.395 (4)
C6—S1	1.715 (3)	C23—C24	1.395 (4)
C7—C8	1.423 (3)	C24—C25	1.387 (4)
C8—C9	1.355 (4)	C24—H24	0.9300
C8—H8	0.9300	C25—C26	1.375 (5)
C9—C11	1.468 (4)	C25—C29	1.441 (5)
C9—S1	1.727 (3)	C26—C27	1.379 (5)
C10—H10A	0.9600	C26—H26	0.9300
C10—H10B	0.9600	C27—C28	1.379 (4)
C10—H10C	0.9600	C27—H27	0.9300
C11—C12	1.384 (4)	C28—H28	0.9300
C11—C16	1.389 (4)	C29—N1	1.126 (4)
			
F1—C2—F2	106.8 (5)	C12—C11—C16	119.0 (3)
F1—C2—C1	115.7 (5)	C12—C11—C9	120.8 (3)
F2—C2—C1	111.1 (5)	C16—C11—C9	120.2 (3)
F1—C2—C3	109.8 (6)	C13—C12—C11	121.1 (3)
F2—C2—C3	108.3 (6)	C13—C12—H12	119.5
C1—C2—C3	105.0 (4)	C11—C12—H12	119.5
F4—C3—F3	106.9 (4)	O1—C13—C14	125.5 (3)
F4—C3—C4	111.4 (5)	O1—C13—C12	114.9 (3)
F3—C3—C4	107.3 (5)	C14—C13—C12	119.6 (3)
F4—C3—C2	115.7 (6)	C13—C14—C15	119.8 (3)
F3—C3—C2	110.6 (5)	C13—C14—H14	120.1
C4—C3—C2	104.7 (5)	C15—C14—H14	120.1
F5—C4—F6	106.4 (4)	C14—C15—C16	121.4 (3)
F5—C4—C5	116.0 (6)	C14—C15—H15	119.3
F6—C4—C5	111.9 (5)	C16—C15—H15	119.3
F5—C4—C3	111.8 (5)	C15—C16—C11	119.1 (3)
F6—C4—C3	108.4 (6)	C15—C16—H16	120.5
C5—C4—C3	102.3 (4)	C11—C16—H16	120.5
F2'—C2'—F1'	102.3 (18)	O1—C17—H17A	109.5
F2'—C2'—C1	112.5 (15)	O1—C17—H17B	109.5
F1'—C2'—C1	103.8 (14)	H17A—C17—H17B	109.5
F2'—C2'—C3'	118.5 (18)	O1—C17—H17C	109.5
F1'—C2'—C3'	113.2 (17)	H17A—C17—H17C	109.5
C1—C2'—C3'	105.8 (11)	H17B—C17—H17C	109.5
F4'—C3'—F3'	106.7 (13)	C19—C18—C22	129.6 (3)
F4'—C3'—C4'	118.9 (17)	C19—C18—S2	110.3 (2)
F3'—C3'—C4'	116.3 (19)	C22—C18—S2	120.0 (2)
F4'—C3'—C2'	104.6 (18)	C18—C19—C20	112.7 (2)
F3'—C3'—C2'	106.0 (16)	C18—C19—C5	125.0 (2)
C4'—C3'—C2'	102.8 (16)	C20—C19—C5	122.3 (2)
F5'—C4'—F6'	101.1 (13)	C21—C20—C19	113.8 (2)
F5'—C4'—C5	108 (2)	C21—C20—H20	123.1
F6'—C4'—C5	107 (2)	C19—C20—H20	123.1
F5'—C4'—C3'	116 (2)	C20—C21—C23	127.3 (3)
F6'—C4'—C3'	110 (2)	C20—C21—S2	110.0 (2)
C5—C4'—C3'	113.5 (15)	C23—C21—S2	122.7 (2)
C5—C1—C7	129.3 (2)	C18—C22—H22A	109.5
C5—C1—C2'	110.0 (9)	C18—C22—H22B	109.5
C7—C1—C2'	120.6 (9)	H22A—C22—H22B	109.5
C5—C1—C2	111.0 (4)	C18—C22—H22C	109.5
C7—C1—C2	119.5 (4)	H22A—C22—H22C	109.5
C1—C5—C19	129.6 (2)	H22B—C22—H22C	109.5
C1—C5—C4'	103.5 (10)	C28—C23—C24	118.2 (3)
C19—C5—C4'	126.8 (9)	C28—C23—C21	120.1 (3)
C1—C5—C4	112.9 (3)	C24—C23—C21	121.6 (3)
C19—C5—C4	117.5 (4)	C25—C24—C23	120.2 (3)
C7—C6—C10	129.4 (2)	C25—C24—H24	119.9
C7—C6—S1	110.49 (19)	C23—C24—H24	119.9
C10—C6—S1	120.1 (2)	C26—C25—C24	120.8 (3)
C6—C7—C8	112.6 (2)	C26—C25—C29	120.0 (3)
C6—C7—C1	123.7 (2)	C24—C25—C29	119.2 (4)
C8—C7—C1	123.7 (2)	C25—C26—C27	119.6 (3)
C9—C8—C7	113.7 (2)	C25—C26—H26	120.2
C9—C8—H8	123.1	C27—C26—H26	120.2
C7—C8—H8	123.1	C28—C27—C26	120.1 (3)
C8—C9—C11	129.6 (3)	C28—C27—H27	119.9
C8—C9—S1	110.2 (2)	C26—C27—H27	119.9
C11—C9—S1	120.3 (2)	C27—C28—C23	121.1 (3)
C6—C10—H10A	109.5	C27—C28—H28	119.5
C6—C10—H10B	109.5	C23—C28—H28	119.5
H10A—C10—H10B	109.5	N1—C29—C25	178.8 (5)
C6—C10—H10C	109.5	C13—O1—C17	117.6 (3)
H10A—C10—H10C	109.5	C6—S1—C9	92.97 (13)
H10B—C10—H10C	109.5	C18—S2—C21	93.21 (13)
			
F1—C2—C3—F4	94.8 (6)	C3—C4—C5—C1	−16.5 (4)
F2—C2—C3—F4	−21.6 (6)	F5—C4—C5—C19	44.6 (5)
C1—C2—C3—F4	−140.3 (5)	F6—C4—C5—C19	−77.6 (5)
F1—C2—C3—F3	−26.9 (6)	C3—C4—C5—C19	166.6 (3)
F2—C2—C3—F3	−143.2 (5)	F5—C4—C5—C4'	−100 (4)
C1—C2—C3—F3	98.0 (5)	F6—C4—C5—C4'	138 (5)
F1—C2—C3—C4	−142.2 (4)	C3—C4—C5—C4'	22 (4)
F2—C2—C3—C4	101.5 (5)	C10—C6—C7—C8	−178.1 (3)
C1—C2—C3—C4	−17.3 (6)	S1—C6—C7—C8	0.5 (3)
F4—C3—C4—F5	−89.5 (6)	C10—C6—C7—C1	2.5 (4)
F3—C3—C4—F5	27.2 (7)	S1—C6—C7—C1	−178.9 (2)
C2—C3—C4—F5	144.8 (6)	C5—C1—C7—C6	48.2 (4)
F4—C3—C4—F6	27.5 (7)	C2'—C1—C7—C6	−127.5 (10)
F3—C3—C4—F6	144.1 (5)	C2—C1—C7—C6	−127.0 (4)
C2—C3—C4—F6	−98.3 (6)	C5—C1—C7—C8	−131.2 (3)
F4—C3—C4—C5	145.8 (5)	C2'—C1—C7—C8	53.2 (11)
F3—C3—C4—C5	−97.5 (5)	C2—C1—C7—C8	53.6 (5)
C2—C3—C4—C5	20.0 (5)	C6—C7—C8—C9	−1.5 (3)
F2'—C2'—C3'—F4'	21.7 (19)	C1—C7—C8—C9	177.9 (3)
F1'—C2'—C3'—F4'	141.4 (14)	C7—C8—C9—C11	−177.7 (3)
C1—C2'—C3'—F4'	−105.6 (16)	C7—C8—C9—S1	1.9 (3)
F2'—C2'—C3'—F3'	−91 (2)	C8—C9—C11—C12	150.9 (3)
F1'—C2'—C3'—F3'	28.8 (18)	S1—C9—C11—C12	−28.6 (4)
C1—C2'—C3'—F3'	141.8 (14)	C8—C9—C11—C16	−29.7 (4)
F2'—C2'—C3'—C4'	146.6 (15)	S1—C9—C11—C16	150.8 (2)
F1'—C2'—C3'—C4'	−93.7 (14)	C16—C11—C12—C13	−0.3 (4)
C1—C2'—C3'—C4'	19.3 (17)	C9—C11—C12—C13	179.1 (3)
F4'—C3'—C4'—F5'	−140.1 (16)	C11—C12—C13—O1	178.9 (3)
F3'—C3'—C4'—F5'	−10 (3)	C11—C12—C13—C14	−1.2 (5)
C2'—C3'—C4'—F5'	105 (2)	O1—C13—C14—C15	−178.3 (3)
F4'—C3'—C4'—F6'	−26 (3)	C12—C13—C14—C15	1.8 (5)
F3'—C3'—C4'—F6'	104 (2)	C13—C14—C15—C16	−0.9 (5)
C2'—C3'—C4'—F6'	−141.1 (17)	C14—C15—C16—C11	−0.6 (5)
F4'—C3'—C4'—C5	94 (2)	C12—C11—C16—C15	1.1 (4)
F3'—C3'—C4'—C5	−136.5 (16)	C9—C11—C16—C15	−178.2 (3)
C2'—C3'—C4'—C5	−21.2 (16)	C22—C18—C19—C20	−177.6 (3)
F2'—C2'—C1—C5	−141.7 (14)	S2—C18—C19—C20	0.3 (3)
F1'—C2'—C1—C5	108.5 (15)	C22—C18—C19—C5	4.4 (5)
C3'—C2'—C1—C5	−10.9 (15)	S2—C18—C19—C5	−177.7 (2)
F2'—C2'—C1—C7	35 (2)	C1—C5—C19—C18	42.1 (4)
F1'—C2'—C1—C7	−75.1 (15)	C4'—C5—C19—C18	−133.1 (7)
C3'—C2'—C1—C7	165.6 (8)	C4—C5—C19—C18	−141.5 (3)
F2'—C2'—C1—C2	15 (61)	C1—C5—C19—C20	−135.7 (3)
F1'—C2'—C1—C2	−95 (63)	C4'—C5—C19—C20	49.1 (8)
C3'—C2'—C1—C2	146 (63)	C4—C5—C19—C20	40.6 (4)
F1—C2—C1—C5	129.1 (6)	C18—C19—C20—C21	−1.4 (3)
F2—C2—C1—C5	−108.9 (5)	C5—C19—C20—C21	176.7 (2)
C3—C2—C1—C5	7.9 (6)	C19—C20—C21—C23	−175.1 (2)
F1—C2—C1—C7	−54.9 (7)	C19—C20—C21—S2	1.8 (3)
F2—C2—C1—C7	67.1 (7)	C20—C21—C23—C28	−28.5 (4)
C3—C2—C1—C7	−176.0 (3)	S2—C21—C23—C28	155.0 (2)
F1—C2—C1—C2'	105 (62)	C20—C21—C23—C24	148.8 (3)
F2—C2—C1—C2'	−133 (62)	S2—C21—C23—C24	−27.7 (4)
C3—C2—C1—C2'	−16 (62)	C28—C23—C24—C25	−0.7 (4)
C7—C1—C5—C19	6.8 (5)	C21—C23—C24—C25	−178.0 (3)
C2'—C1—C5—C19	−177.1 (9)	C23—C24—C25—C26	0.2 (5)
C2—C1—C5—C19	−177.6 (4)	C23—C24—C25—C29	178.8 (3)
C7—C1—C5—C4'	−177.1 (6)	C24—C25—C26—C27	0.6 (5)
C2'—C1—C5—C4'	−1.1 (11)	C29—C25—C26—C27	−178.0 (3)
C2—C1—C5—C4'	−1.6 (7)	C25—C26—C27—C28	−0.9 (5)
C7—C1—C5—C4	−169.6 (3)	C26—C27—C28—C23	0.4 (5)
C2'—C1—C5—C4	6.4 (10)	C24—C23—C28—C27	0.4 (4)
C2—C1—C5—C4	5.9 (5)	C21—C23—C28—C27	177.8 (3)
F5'—C4'—C5—C1	−115.1 (14)	C14—C13—O1—C17	−2.6 (5)
F6'—C4'—C5—C1	136.6 (12)	C12—C13—O1—C17	177.3 (3)
C3'—C4'—C5—C1	15.1 (13)	C7—C6—S1—C9	0.5 (2)
F5'—C4'—C5—C19	61.1 (15)	C10—C6—S1—C9	179.3 (2)
F6'—C4'—C5—C19	−47.2 (15)	C8—C9—S1—C6	−1.4 (2)
C3'—C4'—C5—C19	−168.7 (9)	C11—C9—S1—C6	178.2 (2)
F5'—C4'—C5—C4	101 (5)	C19—C18—S2—C21	0.6 (2)
F6'—C4'—C5—C4	−7(3)	C22—C18—S2—C21	178.7 (2)
C3'—C4'—C5—C4	−129 (5)	C20—C21—S2—C18	−1.4 (2)
F5—C4—C5—C1	−138.4 (4)	C23—C21—S2—C18	175.7 (2)
F6—C4—C5—C1	99.4 (5)		
